# Propagating shocks and transitions across networked food systems: Insights from multiplayer microeconomic modeling

**DOI:** 10.1073/pnas.2321155123

**Published:** 2026-10-26

**Authors:** Benjamin F. Zaitchik, Sauleh Siddiqui, Belay Simane, Ying Zhang, Sriram Sankaranarayanan, Craig Bakker, Steven Witkin

**Affiliations:** ^a^ Department of Earth and Planetary Sciences, Johns Hopkins University, Baltimore, MD 21218; ^b^Department of Environmental Science, American University, Washington, DC 20016; ^c^ Center for Environment and Development Studies, College of Development Studies, Addis Ababa University, P.O. Box 1176 Addis Ababa, Ethiopia; ^d^ Joint Global Change Research Institute, Pacific Northwest National Laboratory, College Park, MD 20740; ^e^ Operations Management, Indian School of Business, Mohali 140306, India; ^f^ National Security Directorate, Pacific Northwest National Laboratory, Richland, WA 99352; ^g^ Department of Civil and Systems Engineering, Johns Hopkins University, Baltimore, MD 21218

**Keywords:** food systems, climate, economic model

## Abstract

Accelerating global change is affecting coupled natural ecosystems and human activities in every part of the world. The local and regional expressions of these changes are a product of interacting and networked systems such that a shock, trend or development transition in one region, ecosystem, or sector can propagate impacts to other regions, ecosystems, and sectors. Informed adaptation and development strategies require an understanding of how signals propagate through these coupled systems and regions. Here, we synthesize findings from our work in Ethiopia and Mali, where we developed and applied a modeling system designed to capture cross-sector dynamics of networked food, water, and energy systems at subnational scale. We approach the problem using a multiplayer microeconomic model that accounts for diverse objectives through chains of food production, distribution, and consumption that connect resource regions to market regions. This paper draws lessons from three case studies in Ethiopia and one in Mali that are designed to examine impacts of a subnational climate shock (drought), a centralized national policy decision (export restrictions in response to food shortage), a distributed development strategy (solar-powered groundwater irrigation), and an external intervention (food aid). In each case, we consider how an initial signal propagates through the country via markets, infrastructure, and interactions between economic actors. An understanding of this propagation can inform strategies for food security in a changing climate.

Global change, including climate and other environmental change as well as the rapid evolution of socioeconomic systems and trade networks, is transforming resource dynamics across scales. The behavior of these complex, interacting systems is poorly understood. Indeed, the role of climate variability in some of the most destabilizing events of recent decades—the 2008 food price crisis (1, 2) or the Syrian civil war (3, 4), for example—is still hotly debated. As climate variability (5) and resource scarcities (6) increase in many regions, we can anticipate that the intensity of these poorly characterized dynamics will increase. And as governments and nongovernment entities move to anticipate and respond to potentially destabilizing shocks, we are likely to see more debates over unintended consequences of development, trade, and investment strategies meant to secure resources and well-being for a particular set of actors.

In this era of change, the stability of food systems and the connected sectors on which they depend is an area of particular concern. Agricultural production patterns are shifting, demanding rapid adaptation in production processes (7, 8), food price volatility has increased (9, 10), populations are moving and urbanizing (11, 12), and economic inequality may be increasing due to climate change (13). Individual components of these dynamics have been the subject of extensive analysis and modeling efforts, including continued improvements in predictive crop yield models at global and local scales (14–17) and considerable efforts to simulate food trade and price dynamics at global scale (18, 19). However, linking these dynamics across scales, sectors, and systems in response to changing shocks and transitions remains an understudied area.

The resolution of processes and the approach to addressing interactions in these models depend on a number of factors, including the targeted scale of analysis. For highly localized studies, for example, agent-based models are sometimes employed that simulate the influence that an individual farmer’s decisions can have on crop production, and how that plays through to food security at the scale of a village or network of settlements. For global scale models, in contrast, food production may be estimated using broad regional yield functions, with a focus on how a price signal or macroeconomic policy can affect flows of trade.

The intermediate scale of regions within a country presents its own set of challenges. At this scale, one may be concerned with the ways that a climate signal impacts various actors in the food economy; or one might be concerned with how a policy or investment made at national scale might influence economic returns and nutritional outcomes across different provinces or states within the country. This is also a scale at which infrastructure can be resolved: Where a global or a nationally oriented country-level analysis might treat transportation, energy distribution, or hydrological flows as generalized abstractions, a model that addresses subnational dynamics will need to consider how specific infrastructure investments and performances influence the distribution of outcomes. This scale of subnational to national analysis is also, we contend, a particularly critical scale for predictive understanding of food systems. It is, in most cases, the primary scale for policy making and for large-scale investments, and it is also the political scale at which flows of people and resources are most likely to be constrained. The most consequential decisions made for adaptation of food systems to global change will likely be made at the scale of the state or the nation.

Here, we engage with the challenges and opportunities for food system modeling at this subnational to national scale through insights from the Distributed Extendible COmplementarity [DECO; (20)]. We have developed and implemented DECO over the past decade in collaboration with colleagues and government officials in Ethiopia, in an effort to provide predictive simulations of multiplayer dynamics within the food system at subnational scale. We present three studies in Ethiopia, each of which considers a different type of shock or transition to the food system that is directly or indirectly related to climate variability: a regional drought affecting food production, a change in national export policy that alters food market dynamics, and a widespread investment in groundwater irrigation systems that increases food production (Fig. 1). To show generalizability, we also present a preliminary translation of DECO to Mali to study a scenario for food aid. The studies in Ethiopia have been reported in detail in previous papers that focused on the specifics of the case and the model developments required to capture relevant dynamics. Those technically oriented papers were of interest for specific policy cases in Ethiopia. In this perspectives piece, we reflect on broader lessons learned from this work and consider their relevance for networked food systems modeling at subnational scale in other contexts.

**Fig. 1.**
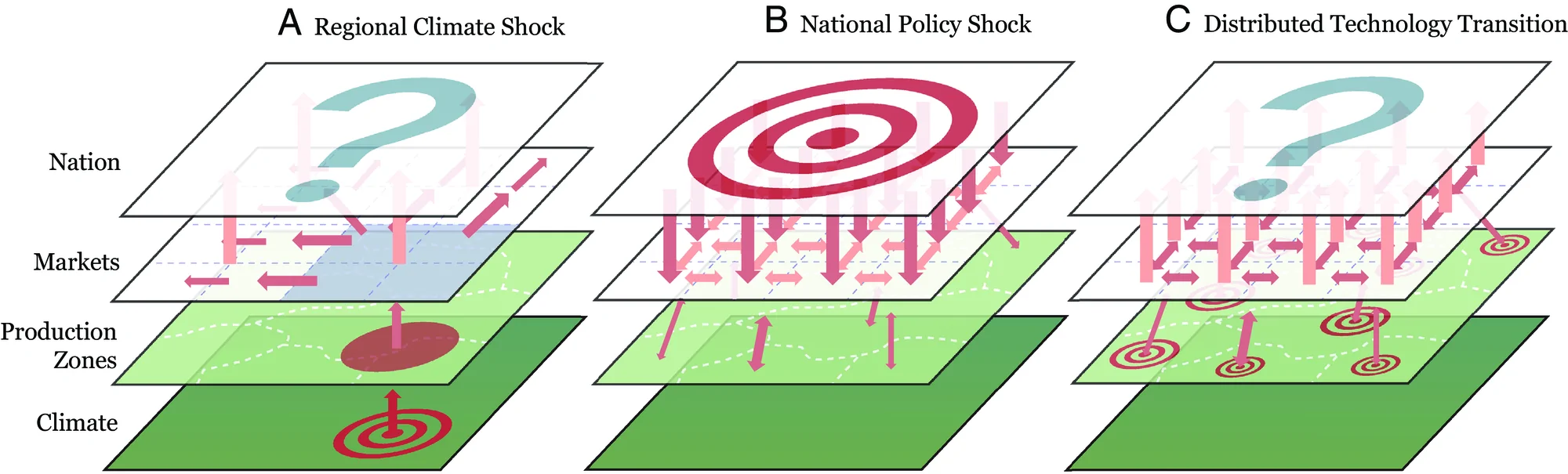
Propagating shocks and transitions across networked food systems. (*A*) A climate anomaly that affects production and can propagate through food markets, with the potential to trigger a national policy response. (*B*) A top–down shock caused by a change at national level—in our case, a policy change, though an exogenous shock from global trade could have a similar dynamic. (*C*) A distributed technology-related transition, in which widespread introduction of a beneficial technology (in our case, a technology that benefits producers) can be communicated via markets and have aggregate impacts at national scale, possibly triggering policy responses or altering food markets beyond national borders.

The first three case studies presented here all focus on Ethiopia. This is the country that DECO was developed in, and given the data-intensive nature of modeling networked systems at this scale it is still the only country with a mature implementation of the model. We have successfully ported DECO to one other country, Mali, for a preliminary implementation which we present as a fourth case study, and we feel that the structure of the model and considerations presented in these Ethiopia case studies are relevant to subnational food system modeling in any developing economy with significant internal food production and sensitivity to food system shocks and transitions.

## The DECO Model

The DECO is a multiplayer microeconomic partial equilibrium model. It is designed to simulate the responses of food markets within a country to potential external shocks and exogenous transitions. DECO simulates multiple aggregate market actors (“players”) in the food system, including crop producers, distributors, storage managers, and consumers. Players seek to maximize their aggregate profit or utility in a noncooperative manner, and the model solves for equilibrium between supply and demand for every exchange between players with explicit representation of production, storage, and transport processes and infrastructure. The model is mathematically represented as a mixed complementarity problem. Such partial-equilibrium models have long been applied in food systems analysis, but DECO is distinctive in its focus on decision-making processes for multiple interacting actors at subnational scale, where each actor is a representative player for their defined role in the food system and geographic unit.

**Fig. 2.**
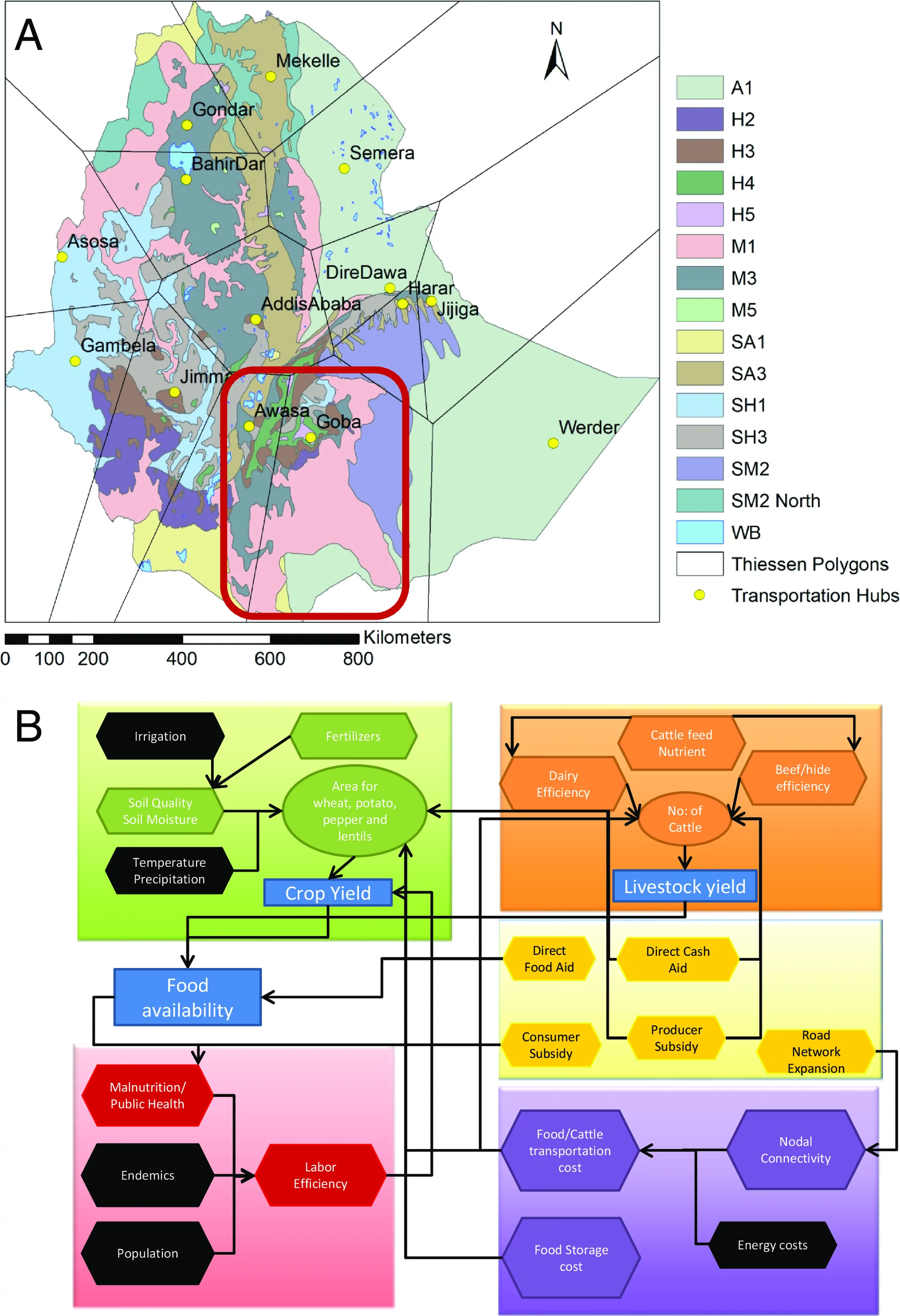
Methodology for representing networked and regional food systems across natural ecosystems and human activity. (*A*) Agroecological zones, transportation hubs, and market catchment areas (Thiessen polygons) in DECO, as applied in refs. 22, and 23. A = arid, SA = semiarid, H = humid, SH = subhumid, M = moist, WB = water bodies. The red rectangle shows the general region (corresponding to eastern Oromia State) that experienced simulated crop failure in Bakker et al. (Note: ref. 20 used an earlier version of DECO with simplified spatial structure.) (*B*) Simplified DECO multiplayer flowchart showing interactions relevant to the case of crop failure response studied in ref. 20.

Spatially, DECO subdivides the country into units relevant for food production and units relative to trade and consumption. In application to Ethiopia, food production is disaggregated on the basis of agro-climatic adaptation zones (Fig. 2) defined by the Ethiopian government (21). Food distribution and exchange is defined using selected transportation hubs connected by the national road network. Production and distribution units are intersected using Thiessen polygons, such that the representative crop producer in any defined production zone sells to one or more markets proportionally based on Thiessen polygon overlap. A similar setup was used in Mali.

The scenarios presented in this paper are drawn from studies that use slightly different versions of the model, and we refer the reader to the original papers (20, 22, 23) for relevant details. Shared characteristics include the fact that crop producers determine land allocation to different primary food crops (teff, sorghum, barley, maize, and wheat), secondary food crops (vegetables) and cash crops (coffee and oil seeds) in order to maximize profit, accounting for expected yield, producer price (the price the crop producer sells to the distributor), and the costs of production including a penalty term for switching crop types in the same farmland over growing seasons. The distributor at a transportation hub then distributes purchased crops to the spatially connected markets, according to price signals of supply and demand. The distributor bears transportation costs and profits from differences in local market prices. All crop prices, including the producer and the consumer price, are endogenously solved at each food market. DECO also includes an external node for international import and export. International import (export) occurs only when the domestic crop price at this node is higher (lower) than the exogenously defined sum of global price and the import (export) cost. The core governing equations and parameters of the model are included here in Supplementary Material.

DECO is calibrated against historical data obtained from the Ethiopian Central Statistical Agency and other sources. Production takes place in Ethiopia’s two dominant growing seasons, “meher” and “belg.” DECO assumes that all produced, nonspoiled crops are consumed, and that the quantity demanded is proportional to the population in the Thiessen polygon surrounding each transportation hub. Price elasticity of demand is derived from ref. 24 and used to estimate the food demand curves for food markets. Food transport quantities are calculated based on the supply and demand at each hub. The model is calibrated by adjusting crop production costs and the slope and intercept of the demand curves. Details for each case study are provided in the corresponding papers (20, 22, 23).

Weather, policy, and development scenarios are then applied to the calibrated model, as described for each case below. In each case, we interpret simulation results as indicative of food system dynamics in response to the exogenously defined pressure. At this stage of development DECO is still a research model, appropriate for advancing understanding of system behavior, rather than a precise policy design tool.

### Case 1: Propagation of a Localized Climate Shock.

One form of climate-related food system shock is an acute disruption of food production due to a climate extreme. A loss of production in one region will have direct impacts on income for food producers in that region and on the local balance of supply and demand. But the shock can also have cascading impacts on other components of the economy, including by affecting food prices and nutritional security in regions that are not directly impacted by the climate extreme. Drought is a relevant example of such a shock. In Ethiopia, a drought-prone country that suffers from chronic and acute food insecurity, a drought-related drop in production in one food-producing region can have market effects that ripple across the country.

To study these dynamics, we implemented DECO for a case of a 25% crop failure in the south of the country, corresponding to the eastern portion of Oromia State (Fig. 2). The character of this crop failure is consistent spatially with previous droughts that have affected the country (e.g., CSA, 2015/2016). As expected, imposing this loss of production had a negative impact on caloric intake in the region to which the shock was applied: DECO simulates that in the month of greatest drought-induced food shortage caloric consumption in the region dropped by over 2% (Table 1), with a 4% drop, on average, for adult females in low-income households. Interestingly, impacts on caloric intake in Addis Ababa were just as severe as they were in eastern Oromia—on the order of 100 kcal per day, on average—reflecting the capital city’s strong trade links with the neighboring Oromia agricultural regions. Food prices and the shadow price of land (indicative of production deficits) and transport (indicative of the demand to move food between regions) were elevated to varying extents throughout the country. In sum, DECO was able to simulate cascading impacts of a localized climate shock across the country, including their impacts on different food staples and different economic and demographic groups, as calibrated using government food system and market data (20).

**Table 1.** Impacts of crop failure and simulated interventions on caloric intake and market prices in eastern Oromia State

Simulation	Intervention	2 y average change in caloric intake	Max. change in monthly caloric intake	2 y average change in cereal price	2 y average change in vegetable price
No intervention	-	−1.1%	−2.3%	17.4%	12.0%
Food aid	30 million kg cereals delivered to eastern Oromia markets each month	−0.5%	−0.5%	-16.4%	11.5%
Cash aid	$7 per person per month to the poorest 50% of the population for the entire country	−0.2%	−0.8%	13.0%	17.8%
Consumer subsidy	20% subsidy of consumer purchases of cereals in eastern Oromia	−0.9%	−1.9%	21.6%	12.0%
Producer subsidy	10% subsidy to producers for cereals grown in eastern Oromia	−1.0%	−2.1%	13.0%	12.3%
Transportation expansion	Maximum interstate bilateral transportation capacity doubled everywhere	−2.2%	−3.4%	44.1%	17.0%

All values are relative to a baseline simulation with no crop failure. Two-year averages are for the year of the crop failure and the subsequent year. Caloric intake values are for the average adult.

Capturing these dynamics allows us to apply DECO to simulate the expected impact of various government interventions on food prices and caloric satisfaction in the wake of the crop failure. In ref. 20, we considered a number of commonly proposed food security interventions: direct food aid, direct cash aid, consumer subsidies, producer subsidies, and improved transportation, intended to ease bottlenecks for food import to the affected region. Each intervention was applied at the scale of a $2 billion investment (Table 1), either directly into eastern Oromia or spread over the entire country. Simulations indicate that direct food and cash aid both have the potential to minimize caloric shortfall in the region, though they do so through very different mechanisms: Direct food aid in the form of cereals directly satisfies demand and stabilizes market prices, while direct cash aid drives prices up through market effects, providing a benefit to producers and, in our simulations, improving dietary diversity. In our simulations, consumer and producer subsidies were not particularly effective at reducing hunger. This is because both of these subsidies are ultimately intended to increase production, either by increasing demand (through increased effective purchasing power) or reducing the cost of production. This can be effective when conditions allow for production to increase, but in our simulations there are strong constraints on production in Ethiopia: Arable land is limited, and transformative agricultural inputs are not readily available. These are realistic constraints for an acute food shortage in the country today, though complementary agricultural development policies could relax those constraints in the future.

Finally, we were initially surprised by the negative impact that increased maximum transportation capacity had on food security. On reflection, however, the result makes sense. Reducing transport constraints has the effect of speeding food to the markets with greatest consumer power—including export markets—and this can have a negative impact on food insecure consumers in regions of supply deficit. Under baseline conditions, transportation was a bottleneck on food producers in eastern Oromia, limiting their access to lucrative markets and suppressing food prices in local markets of this poor but agriculturally important region. When that bottleneck is relaxed by increasing the maximum possible transport capacity, food flows out of the region despite local shortages that affect the food insecure. Benefits to food insecure populations in other regions are limited, since market dynamics drive the food to more secure (i.e., wealthier) consumers and to export.

A key methodological consideration for this result is the representation of the food transportation network. The result emerges from the linked dynamics present in the model; it would not be present in an analysis that simply considered national supply and demand, or that used local crop yield as a proxy for local food availability. The fact that transportation serves to communicate the impacts of drought shock, but that an expansion of maximum transportation capacity ends up exacerbating local impacts rather than dissipating them, is very much a product of the structure of the case study. We have tested for acute response to a single shock. In that context, transport is simply a valve on free trade. In reality, long-term investment in transportation infrastructure will have a range of impacts on economic and social development, including a number of food system dynamics. These realities could be incorporated into the model, as informed through a participatory development process.

The results of this simulation are certainly not intended to suggest that Ethiopia should not invest in transportation infrastructure. Rather, they highlight the importance of considering networked markets and distributional impacts during a climate-induced reduction in food supply, and in particular the potential for market integration to create vulnerability to exogenous shocks (25). The seemingly counterintuitive result that enhanced transport reduces local food security is logical when considered in the context of market dynamics.

### Case 2: Distributed Impacts of a Centralized Policy Decision.

In response to commodity price shocks of recent years, many countries have considered or implemented export restrictions on staple crops in order to safeguard against domestic food shortages (26). The impacts of these restrictions on global food markets have been the subject of extensive study (27), with particular focus on the potential for such restrictions to set off a spiral of speculation and crop hoarding that can dramatically exacerbate even a modest initial perturbation to global food supply. A complementary, less-studied question is how the policy impacts markets and food outcomes within the country that implements the export ban.

In the wake of the 2008 global commodity price crises, Ethiopia implemented a ban on the export of teff, a grain that is a critical staple of many Ethiopian diets. The ban was motivated both by the acute shock of the 2008 price crisis and by the recognition that teff, a gluten-free grain with high nutrient content, was gaining popularity as a “superfood” in wealthy countries, potentially driving a long-term trend in external demand for the crop. This ban stood for several years before being partially lifted in 2015. This form of export control policy is intended to protect food security, but it is recognized that it comes at a potential cost of lost revenue in the agricultural sector. DECO allows us to study how those benefits and costs might be expected to be distributed across regions and through the food supply chain.

In ref. 22, we probed these dynamics by studying the sensitivity of food production, markets, and consumption across the country under conditions of a total ban on teff exports and several relaxation scenarios: i) allowing export of up to 200,000 quintals of teff per year; ii) as in (i) but also projecting a 10% increase in global demand; iii) allowing export of up to 2 million quintals of teff each year; iv) as in (iii) but also projecting a 10% increase in global demand; and v) allowing a fully free market for teff export. We were specifically interested in whether these various policies would impact domestic regional food consumption patterns and in how any economic benefits of a freer global teff market would be realized by different players within the domestic food system. We note that DECO is a microeconomic model, such that results of these simulations should be interpreted as estimates of the direct impacts of an export policy change propagating through the Ethiopian food system, rather than as a projection of a new equilibrium after potential policy adjustments or macroeconomic changes in Ethiopia or elsewhere.

**Fig. 3.**
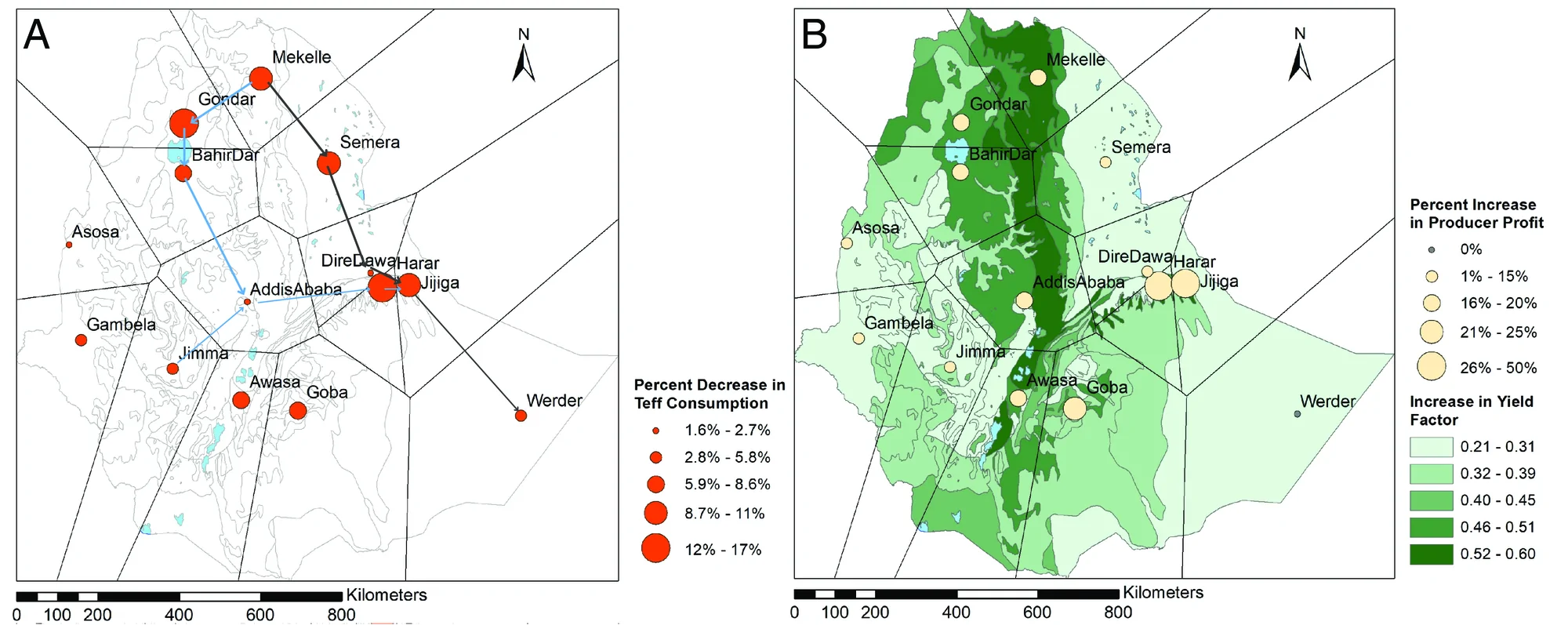
(*A*) For Case 2, the impact of full removal of the teff export ban on teff consumption in each major market area (circles) and on transport of teff between regions (arrows; blue = increase, gray = decrease, thickness proportional to magnitude of change). (*B*) For Case 3, the increase in maize yield factor when groundwater irrigation is available (green shading) and the percent increase in producer profit from irrigation (circles).

Applying DECO to study the propagating impacts of a relaxation on the teff export ban allowed us to add nuance to the narrative that liberalizing exports would benefit domestic teff producers simply by virtue of increased demand (28–30). In our simulations, relaxing the ban does substantially increase export revenue for Ethiopia: in the case of a 2 million quintal cap, we estimate that teff exports at $160 million per year, which would add 24% to Ethiopia’s noncoffee agricultural export revenue. The total economic benefit to producers, however, was modest, as teff producers tend to be small-scale farmers who lack market power over distribution and storage networks. Instead, the large majority of economic benefits were realized by warehouse operators and distributors who could benefit from the additional export market without competitively passing a share of those benefits to the distributed producers. Simulations also included meaningful, regionally specific impacts on consumption (Fig. 3*A*). Food substitution took place in response to the higher market price of teff, and this effect was particularly pronounced in regions where teff consumption exceeds local production. The increased price of teff propagated to other food staples, and there was, overall, a small reduction in total domestic food consumption when the export ban was relaxed or removed.

Additionally, the lifting of the teff export ban led to a notable shift in teff transport patterns (Fig. 3*A*). Assuming the presence of efficient transportation systems where the cost of delivering food grains is mostly determined by the distance traveled, we notice a higher volume of food being delivered from the fertile areas of Mek’ele, Gondar, and Bahir Dar to Addis Ababa for export, a pattern that is facilitated by an increase in the simulated grain storage capacity in proximity to the capital, which the warehouse operators can utilize to export teff during the off-season, when it is possible to achieve higher prices. As a result, a smaller amount of teff was shipped to market-dependent dry regions in the East of the country (e.g., Semera, Dire Dawa, Werder) since the storage and distribution operators were unable to achieve the same profit margin as they would through teff exports.

An important feature of DECO for this case study is its use of different regional disaggregation for modeling production and consumption of crops (adaptation regions) and transportation of crops (market regions). This allows for granular and computationally efficient representation of the production, transportation, consumption, and export of agricultural goods. This granularity enhances the fidelity of our insights with respect to domestic food systems dynamics and provides a testbed for examining how complementary policies for grain markets and distribution systems might affect the pattern of risks and benefits of a change in export policy. The export driver itself, however, is simplified by the national boundaries of our model. We do not simulate the impact that an increase in global demand for teff will have on other economies and how that could feed back to affect Ethiopia. Such a simulation requires coupling with global trade models that we have not yet implemented.

### Case 3: Large-Scale Implications of a Distributed Development Strategy.

Access to water for irrigation can have a transformative effect on food production. In Ethiopia, the vast majority of agricultural production is rainfed, and it is still largely pursued by smallholder farm households in low-input, low-output mixed crop and livestock systems. Though Ethiopia is known as the “water tower of Africa,” including extensive humid highland areas that receive a surplus of rainfall, these rains are seasonal and subject to considerable interannual variability, and large portions of the country, especially in the north and the east, are semiarid to arid. Centralized surface water irrigation schemes have been implemented, particularly for larger-scale plantation farming, but the distributed character of smallholder agriculture and the dissected topography of the Ethiopian highlands limit the penetration of these centralized surface water schemes. Groundwater irrigation, however, has considerable potential (31–33). Recognizing this potential, rural development schemes in recent years have sought to increase the availability of small-scale, groundwater-fed irrigation systems for smallholder farmers (34).

We refer to potential expansion in implementation of small-scale stand-alone technologies that can be relatively easily distributed without relying on centralized large-scale infrastructure as “distributed development.” In this case study, we specifically refer to small-scale groundwater irrigation development, which is well suited for the predominantly dispersed small farms in Ethiopia. Additionally, compared to centralized large-scale irrigation systems, such as constructing a reservoir for irrigation withdrawal, distributed small-scale irrigation using groundwater requires much smaller investment and thus is more likely to be implemented by smallholder farmers. These small-scale, distributed systems can have direct benefit to producers. As use of such systems scales up, however, there are concerns about impacts on groundwater sustainability, and the nature of economic benefit to producers may change as the technology becomes more widely available. Indeed, the experience of widespread groundwater irrigation pumps in other countries offers reasons for concern for resource sustainability (35).

To assess the risks and benefits of expanded access to groundwater irrigation, we introduced irrigation and groundwater extraction modules to DECO. These modules, coupled to the crop production model within DECO, allow us to simulate the impacts that irrigation has on food production and on water resources, and how the resulting increase in production propagates through the food system. This coupled simulation system can then be applied to study short-term events, such as the impact of an acute drought, and longer-term trends. In ref. 23, we considered the former case and examine how food production and groundwater resources would fare during a major drought like the 2015 event if smallholder farmers had widespread access to small, solar powered groundwater irrigation systems.

In this first application of the coupled water-food system version of DECO, we considered the simplified case of irrigation applied to a single staple crop, maize, and for a scenario in which the penetration of the technology was equal across all maize-producing regions in which shallow groundwater is accessible. We consider location-specific benefits of irrigation by integrating data on climate variability, soil fertility, shallow groundwater availability, and market access across different regions. This resulted in varying levels of crop yield improvement across regions and spatial heterogeneous price dynamics. For example, in fertile, prone-to-drought areas that have better access to markets, the benefit of irrigation is relatively high as water availability can significantly enhance productivity in these areas and farmers can more efficiently sell their surplus produce. We do not consider the cost of implementing irrigation technology in our model; however, our approach could be interpreted as a representation of fully subsidized solar pumps for irrigation, which would likely have minimal regional differences in costs despite spatial variability in groundwater depth, as solar pumps do not incur any fuel costs for pumping the water.

In contrast to the teff export study, the study of small-scale irrigation presents a case of distributed investments in a production technology (Fig. 1*C*). This offers a much more direct path to increasing production and benefiting producers. Indeed, simulations indicate a substantial increase in producer profit attributable to irrigation during the drought year. This benefit was concentrated in regions where water scarcity is the main barrier to growing crops and where the drought was particularly acute (Fig. 3*B*). Total crop production also increases dramatically, driven by the increase in maize, even though there is very little change in total cropped area on account of land constraints. These benefits come at the cost of groundwater withdrawals for irrigation. For this case of a one-year drought, our simulation suggests that these reductions were not so severe that they would be expected to impact the viability of groundwater irrigation at a regional scale. However, over longer time horizons, a continued pattern of more frequent droughts, coupled with an increased reliance on groundwater for irrigation, could potentially lead to significant impacts on water resources. This could, in turn, trigger policy responses that feed back on crop-water dynamics—something that was not relevant to the one-year case study but that could readily be incorporated to DECO.

Interestingly, in our single-year irrigation case study, we do not see meaningful increases in caloric intake associated with increased food production. This is because much of the increased production goes to export—a result that is not surprising, given the economically rational, free market assumptions of DECO and the absence of any complementary protectionist policies in our simulation. This specific result may or may not be realistic, both because complementary policies could well be expected during an acute food shortage like the one that occurred in 2015 and market players might redirect their behavior when the country is in food crisis. Social destabilization, including migration, could also impact the food system, as could the presence of transportation cartels (36, 37) and these dynamics are not yet included in DECO.

This third case study was our most broadly integrated implementation of DECO, as it brought groundwater extraction and explicit simulation of climate sensitivity and water resource dynamics into the model. In interpreting the simulations, then, we are cognizant of several modeling decisions that influence results. These include the incorporation of high-resolution hydroclimate and soil data, detailed parameterization of surface runoff and groundwater recharge across spatially gridded soil columns, calibration to bias-corrected climate and land surface model outputs, and consideration of regional variations in shallow groundwater availability. The resulting soil–water balance model is capable of tracking daily soil moisture changes while simulating runoff and infiltration processes under varying climate and irrigation conditions. It also captures the nuanced interactions between water availability and crop yield, accounting for soil properties and crop characteristics in addition to climate and irrigation conditions, while estimating changes in groundwater storage due to deep percolation and irrigation withdrawals.

### Case 4: Delivering Food Aid and Showing Applicability to Mali.

We now present a case study in Mali to show the translation of DECO to another country. Mali presents a valuable comparison to Ethiopia on account of significant food system similarities and differences. Like Ethiopia, Mali is predominantly rural, subsistence oriented, climate vulnerable, and food insecure. However, Mali is different from Ethiopia in important ways. The Malian population is about 1/6 that of Ethiopia, but is projected to grow at a faster rate. Mali is characterized by weak transportation infrastructure and particularly high rural isolation from markets. Exposure to climate variability is also distinct, as Mali follows uniform Sahelian patterns of precipitation variability, while Ethiopia has multiple rainy seasons and low correlation of rainfall variability across subregions.

While Ethiopia and Mali are both prone to damaging droughts that harm food security, their climate and topography are significantly different. Ethiopian climate zones range from humid tropical alpine to hyperarid with steep topographic and pluvial gradients, while Mali ranges from humid tropical in the South, through the steep meridional precipitation gradient of the Sahel, to arid in the North. Finally, unlike Ethiopia, Mali depends heavily on food imports, that have been rising over the past decade (38). This dependence on imports motivated an application of DECO to study the sensitivity of food prices, and associated food insecurity, across the country in response to a direct infusion of food aid to the import hub at Bamako. In this case study, we use DECO to analyze the impact on consumer surplus at the subregional level in Mali from direct food aid.

**Fig. 4.**
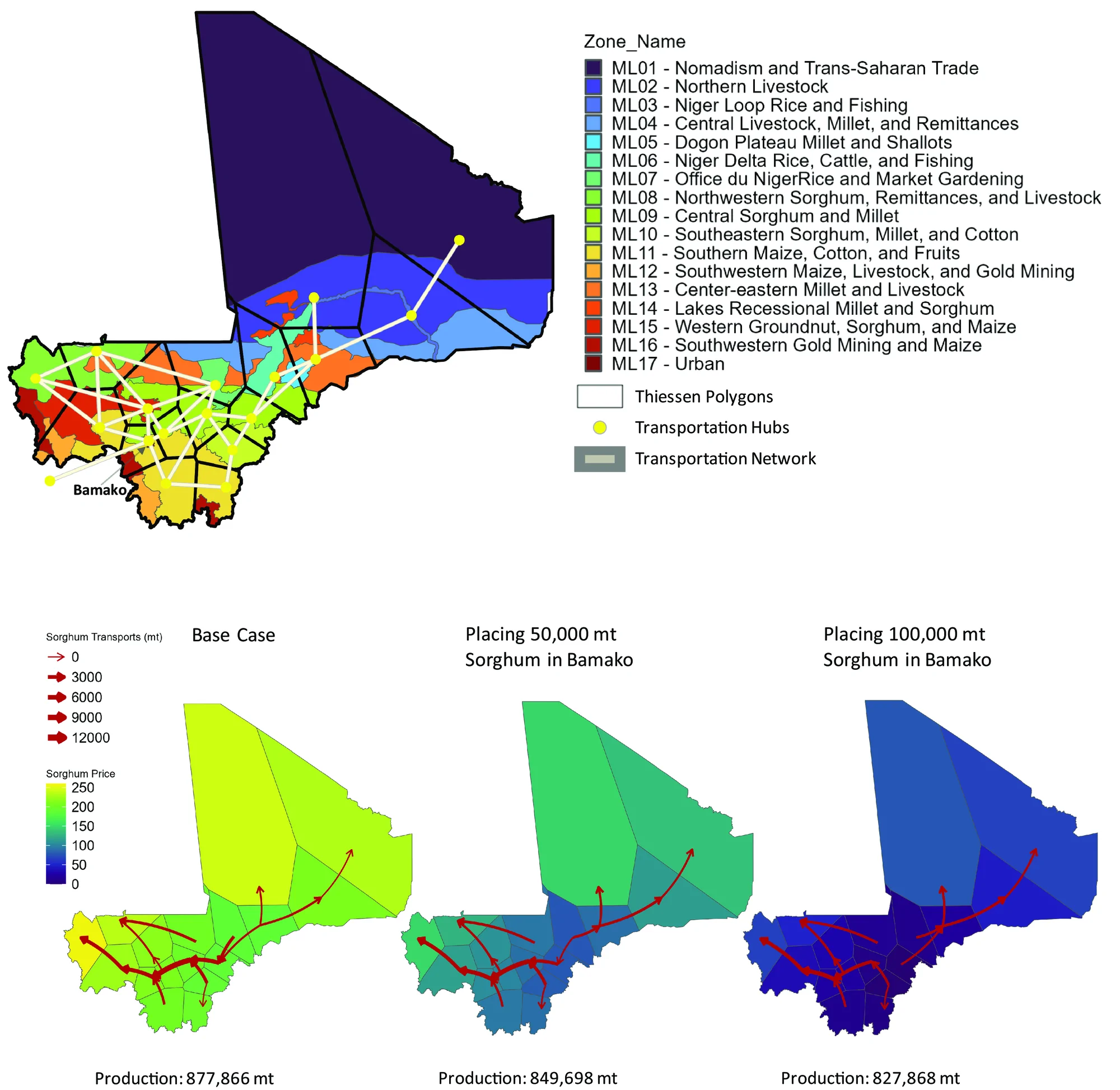
Similar to Ethiopia, we create market polygons and a transportation network for Mali and use Mali Livelihood (ML) zones for food production (*Top*). An introduction of food aid at Bamako alters DECO-simulated food flows across the country (*Bottom*).

We use a similar strategy for regionality in DECO as we did for Ethiopia (Fig. 4, *Top*). For consumption data, we had more detail in Mali compared to Ethiopia since we had access to the Enquête Harmonisée sur le Conditions de Vie des Ménages (EMOP) dataset (http://instat-mali.org/index.php/emop). For this preliminary study, we estimated the total yearly per-capita consumption in kg of food commodities on the commune level in Mali for 2011, as that year has the most spatially complete data. We were also able to estimate demand elasticities from these data, by using price data from the World Food Programme (https://dataviz.vam.wfp.org/). We also combined multiple data sources to estimate crop areas, production, and yields.

Our results show similar insights when we test what the change in consumer surplus and food transportation would be if food aid of either Sorghum or Maize was introduced into the Bamako market at no cost. Results show how consumer surplus and transportation patterns change with the different amount and type of aid introduced (Fig. 4, *Bottom*). As expected, market connectivity allows the influx of food aid sent to Bamako to propagate across the country. In this case study, the influence that aid has on food prices in the food insecure northern parts of the country are not achieved by direct transport from Bamako, but by market forces that redirect trade flows from the grain basket region in the center of the country toward the northeast and, to some extent, the Nioro du Sahel (Nioro) market hub in the West, with reduced flows to Bamako.

The character of these results holds across the food aid scenarios we tested and across sensitivity tests for model parameters. Quantitatively, however, the results are highly sensitive to elasticity in demand and the efficiency of transportation. These sensitivities point to targeted studies that could improve understanding of food price dynamics in Mali in response to food aid programs. First, by explicitly including the transportation network, a model like DECO can be used to identify transportation bottlenecks under different market scenarios and can inform targeted investments to alleviate those bottlenecks. Second, by resolving subnational consumption patterns, DECO makes it possible to integrate space and time-varying elasticity to studies of food price dynamics, accounting for local dietary preferences and practices under conditions of scarcity. Doing so in a meaningful way requires local studies of food production, consumption, and substitution patterns.

## Discussion and Conclusions

Reflecting on the development and applications of DECO to date, we recognize the deep and sometimes daunting uncertainties involved in simulating food systems affected by shocks and transitions. DECO is not a quantitatively precise tool: Like any model of complex natural-human systems, it is sensitive to parameterizations and model formulation decisions. Such models are useful if they allow us to learn something about the dynamics of the systems in question, and they can become actionable if, like DECO, they are codeveloped and evaluated as narrative tools in collaboration with relevant experts and decision-makers. Results from DECO or other models like it should not be interpreted in absolute quantitative terms but through relative, qualitative changes in output given the scenario under question. The parameterization and calibration are designed for one particular baseline case, which should be viewed as a counterfactual that is to be compared against. The absolute output is typically not the focus, but relative changes from the counterfactual can be interpreted consistently.

Each of the four case studies presented here was developed in response to policy or development questions of specific interest in Ethiopia or Mali. Taken together, however, they point to several important features of DECO that generally apply to this form of food systems modeling. First, networked models can yield surprises relative to models that present a non-networked or static view of food security, such as the impact of expanded maximum transportation capacity in Case 1, and that embodying those surprises in a simulation model provides a platform for exploring policy options. While other network models could capture this kind of gating effect, the intermediate scale and integrated multiplayer market structure of DECO resolves the explicit transportation infrastructure of Ethiopia to distinguish between drought-affected and non-drought-affected production regions and their relationship to marketsheds, and to spatially and temporally quantify the potential roles of transport and storage operators to address undesirable outcomes.

Second, these models can usefully demonstrate how policy aspirations may play out across a distributed system. Case 2, for example, shows that the distribution of benefits from relaxed export restrictions is limited by the current state of farmers’ access to open markets. Case 4 shows similar dynamics with the introduction of food aid in a different country. The quantitative details of the result are sensitive to model settings, but the character of the result holds policy relevance. Here, specific aspects of modeling food systems at intermediate resolution come into play. For example, in both Case 2 and Case 4, our results were heavily influenced by elasticities of demand that can vary within populations based on cultural preferences, proximity to production zones, and market options. In these case studies, DECO exhibits substantial sensitivity to uncertain local elasticity parameters, pointing to food system dynamics that require further research. But in both cases, the overall patterns of response to the shock still provided insights. For Case 2, which addressed export policy, the ability to resolve linkages between production zones, consumption marketsheds, and export nodes, all in the context of potential local differences in elasticities, allows us to investigate how the change in policy might influence consumption regions that are involved in neither production nor export, but that can be vulnerable due to dietary preferences. Case 2 shows the value of collecting such elasticity data for quantifying impacts from policies.

Third, the ability of these models to integrate sectors and geographic regions can be informative across systems and infrastructures. As Case 3 shows, a widely discussed rural economic development strategy like solar-powered groundwater irrigation has implications for food security under an acute climate shock and for longer-term water resource sustainability that are mediated by market exchange. DECO offers a flexible simulation tool that can support cross-agency and cross-sector evaluation of these implications. In this respect its role is similar to that of previous decision-oriented systems analysis tools (39, 40), but with a degree of coupled process modeling and resolved interplayer dynamics that was not typically present in such models. This can be particularly critical for food systems because production depends so strongly on the local natural resource base. Source sustainability of groundwater wells—for irrigation or for competing water uses—can be highly localized and have lasting impact, while the market benefits of increased food production are diffuse and, potentially, transient. Resolving local hydrogeology and production zones in the context of a networked food system allows for a more meaningful accounting of benefits and a more physically realistic simulation of potential policy impacts for any food system that includes production under limited resources.

We stress that models such as DECO should be used to generate results that illustrate the implications of different assumptions rather than produce precise predictions. While quantitative assessments of uncertainty can be useful, they can dramatically underestimate true uncertainties associated with model structure and scope. Taking the narrative stance of “what might happen if…” presents the modeling exercise as a process of understanding linked dynamics. This process informs expectations of how a given shock or system change might be expected to influence outcomes. DECO also provides a mathematical structure to explain the results that it produces in terms of the model formulation and parameter values. That structure then enables us to connect model behavior to real-world behavior by tracing the chain of causality and assessing the plausibility of that chain. We can thereby provide results that are robust to poorly calibrated parameters–namely, insight into the real-world counterpart of the model. That insight allows us to construct a narrative about how the modeled scenarios would play out in the real world; the narrative can then inform policy.

DECO is particularly valuable in this regard because the stories it tells include multiple sectors and decision-makers in a scenario: It is sufficiently detailed to trace out the chain of impacts from different hypothetical changes to that scenario. For example, we can see how an increase in irrigation might also require export restrictions, which were not modeled, to significantly improve local food security. This stems from the model’s integration of otherwise siloed understandings of agriculture, resource, and market dynamics. The value of DECO’s flexible structure is exemplified by ongoing work at the Pacific Northwest National Laboratory (PNNL) to model global food security scenarios, where the model has been further developed to relax assumptions about perfect foresight, drastically reduce computational cost, enable automated calibration, and expand the range of scenarios that can be modeled. Lessons learned from our case studies in Ethiopia and translation to Mali are now being applied to the global application of the modeling approach.

As we extend DECO to new capabilities and contexts, we continue to address challenges that were evident in these first case studies. First, DECO is complicated and parameter-heavy. This presents a fundamental challenge to codesign exercises, as participants need to commit to multiple sessions to diagnose model development needs, evaluate utility, iterate on model design, interpret uncertainties, and assess case studies. Striking the balance between detailed realism and lighter, more interactive simulation is a challenge for any attempt to apply complex systems analysis to codesigned policy exercises. This may be particularly true at the intermediate scale of a model like DECO. Because the model includes multiplayer dynamics and resolves infrastructure, it lends itself to policy applications that target specific locations or food system processes. That specificity is a motivating strength of the approach, but it also raises the bar on matching the detail of model applications to the detail of model realism.

Second, systems models like DECO are difficult to evaluate in a convincing way. While components of DECO have been calibrated and evaluated, either by us or by other research groups, it is challenging to evaluate the reliability of the full system for scenario analysis. The food system of any country includes multiple dependencies across scales, making it hard to calibrate and evaluate a full food system model for specific historical events given a limited data record. We can assess the realism of simulations statistically, in terms of representing the mean state and volatility of key components of the food system, and we can assess the directionality, response time, and geographic distribution of some elements of scenario simulations, but considerably more data collection and model development would be required to achieve the event-scale precision that a decision-maker might expect. Given the cultural and geographic specificity of food systems, achieving this precision in a manner that is readily generalizable requires additional care. Our translation from Ethiopia to Mali represents a starting point for understanding model behavior in different countries, but there is more work to be done. Moving the model to a second country has emphasized the challenges of interpreting consumption data that is provided in different forms for different countries, of customizing methods for estimating relationships between climate and food production (precipitation, satellite-derived greenness, and crop yield correlate strongly in the Malian Sahel but not in all zones of Ethiopia), and of matching the temporal discretization of the model to resolve local systems (e.g., one dominant cropping season in Mali, but multiple in Ethiopia).

The challenge of evaluation ties directly to the classic challenge of cascading uncertainties in complex and multiscale systems models (41, 42). It is not a given that uncertainties increase with additional model coupling—it is possible that sensitivities cancel out, or that downstream prediction targets are robust to upstream modeling uncertainties—but there is a general tendency for uncertainty ranges to widen as additional components are introduced to a modeling system (43). This reality can make one question whether something as complex as a national food system is even a reasonable target for predictive modeling. We believe that it is, under certain conditions.

Alongside these cascading uncertainties, there are structural model assumptions that may be particularly acute when modeling something as broadly inclusive and as personal as a food system. DECO is a microeconomic model that assumes rational decision-making at an aggregate regional level. Subrational preferences can be accounted for in model parameterizations, but doing that correctly requires localized information on preferences and behaviors that often cannot be derived readily from standard agriculture and market datasets. For cases of complex subrational dynamics, it may be useful to implement an agent-based model (ABM) at local scale and develop parameter response surfaces to link the ABM to DECO.

Finally, headwinds that our research groups have faced during COVID-19 and the period of political instability in Ethiopia point to the importance of integrating systems modeling efforts to institutional relationships and workstreams that extend beyond standard grant cycles. A committed independent group of researchers and practitioners can make important progress in the development and application of these complex systems models. But once a modeling system is sufficiently mature, it makes sense for further development and application to take place in a stable institutional pipeline. Programs like the Famine Early Warning System Network [FEWS NET; (44)], the NASA/USAID SERVIR program (45), and centers like the International Water Management Institute (IWMI), and many programs within national and state government agencies offer demonstrations of how this pipeline can work. Subnational multiplayer food systems models like DECO are not, to our knowledge, currently in the portfolio of these institutions, but we believe that there is strong potential for the DECO approach to transition from research to a more lasting role in decision support.

## Supplementary Material

Appendix 01 (PDF)

## Data Availability

There are no data underlying this work.
