# Supplementary material for: Propagating shocks and transitions across networked food systems: Insights from multiplayer microeconomic modeling
Source: Proc Natl Acad Sci U S A. 2026 Oct 26;123(44):e2321155123. doi: 10.1073/pnas.2321155123 (PMC13732204; doi:10.1073/pnas.2321155123)
Supplement: Supplementary file 1 [file pnas.2321155123.sapp.pdf]

Propagating shocks and transitions across networked food systems: insights from multiplayer microeconomic modeling: Supplementary Material

Benjamin F. Zaitchik, Department of Earth and Planetary Sciences, Johns Hopkins University, Baltimore, MD, USA

Sauleh Siddiqui, Department of Environmental Science, American University, Washington DC, USA

Belay Simane, College of Development Studies, Addis Ababa University, Addis Ababa, Ethiopia

Ying Zhang, Joint Global Change Research Institute, Pacific Northwest National Laboratory, College Park, MD, USA

Sriram Sankaranarayanan, Indian Institute of Management Ahmedabad, Ahmedabad, India

Craig Bakker, Pacific Northwest National Laboratory, Richland, WA, USA

Steven Witkin, Department of Civil and Systems Engineering, Johns Hopkins University, Baltimore, MD, USA

Corresponding Author: Benjamin F. Zaitchik, zaitchik@jhu.edu, +1-410-516-4223

We describe the generic model formulation discussed in our review paper that is taken directly from Bakker et al., 2018; Sankaranarayanan et al., 2020; and Zhang et al., 2024. Please refer to those papers for the specific formulations and methodologies.

Notation

The table below describes notation for our sets, parameters, and variables.

Supplementary Table 1: Summary table of model parameters and variables.

**Symbol**	**Explanation**	**Symbol type**
y	Year	Set
s	Season	Set
z	Adaptation zone	Set
n	Market	Set
c	Crop	Set
ξ	Animal produce – beef and milk	Set
f	Food (*i.e.*, Crops and animal produce combined)	Set
r	Roads (Connecting two markets s_r and d_r – the source and destination)	Set
R_{in}^{n}, R_{out}^{n}	Set of roads coming into and going out of a market location n	Set
df_y	Discount factor for year y	Parameter
Ψ_{zn}	Fraction of adaptation zone z that is in the Thiessen polygon corresponding to market n	Parameter
$Q_{znfsy}^{F\to D}$	Quantity of food f sold by producer in z to a distributor in market n in the season s of year y	Primal Variable
$\pi_{nfsy}^{D\to F}$	Price for food f paid by a distributor to any producer in market n in the season s of year y	Dual Variable

A_{zcsy}	Area allotted for crop c by the crop producer in z in the season s of year y	Primal Variable
Q^{F}_{zfsy}	Quantity of food f produced in adaptation zone z in the season s of year y	Primal Variable
C^{F}_{zcsy}	Cost of raising unit area of crop c for the crop producer in z in the season s of year y	Parameter
C^{chg}_{zsy}	Penalty for change in cropping patterns between adjacent years	Parameter
Y_{zcsy}	Yield of crop c in adaptation zone z in the season s of year y	Parameter
Q^{C}_{zsy}	Number of cattle reared in adaptation zone z in the season s of year y	Primal Variable
Q^{sl}_{zsy}	Number of cattle slaughtered in the adaptation zone z in the season s of year y	Primal Variable
$Q^{C,\xi}_{zsy}$	Quantity of animal product ξ produced in the adaptation zone z in the season s of year y	Primal Variable
$Q^{C\to D,\xi}_{znsy}$	Quantity of animal product ξ sold by the livestock producer in z to the distributor in market n in the season s of year y	Primal Variable
$\pi^{D\to C,\xi}_{nsy}$	Price for animal product ξ paid by the distributor in market n to any livestock producer in the season s of year y	Dual Variable
C^{C}_{zsy}	Cost of rearing cattle in the adaptation zone z in the season s of year y	Parameter
Y^{ξ}_{zsy}	Yield of animal product ξ in the adaptation zone z in the season s of year y	Parameter
k	Proportion of cattle that should be slaughtered before they die	Parameter
r_{birth}, r_{death}	Birth and death rate of cattle	Parameter

$Q_{zsy}^{C,Herd}$	Minimum heard size to be maintained	Parameter
$Q_{nfsy}^{D_b}$	Quantity of food f brought by distributor in market n in season s of year y	Primal Variable
$Q_{nfsy}^{D_s}$	Quantity of food f sold by distributor in market n in season s of year y	Primal Variable
Q_{rfsy}^{D}	Quantity of food f transported by distributor through road r in season s of year y	Primal Variable
C_{rfsy}^{D}	Cost of transporting food f through road r in season s of year y	Parameter
$\underline{Q_{rsy}^{D}}$	Maximum Quantity of food that can be transported through road r in season s of year y	Parameter
$\pi_{nfsy}^{W\to D}$	Price for food item f paid by a store to a distributor in market n in the season s of year y	Dual Variable
$\pi_{nfsy}^{U\to W}$	Price for food item f paid by a consumer to a store in market n in the season s of year y	Dual Variable
$Q_{nfsy}^{W_b}$	Quantity of food f brought by the store in market n in season s of year y	Primal Variable
$Q_{nfsy}^{W_s}$	Quantity of food f sold by the store in market n in season s of year y	Primal Variable
Q_{nfsy}^{W}	Quantity of food f stored by the store in n in season s of year y	Primal Variable
C_{nfsy}^{W}	Cost of storing food f at n in season s of year y	Parameter
$\underline{Q_{nfsy}^{W}}$	Maximum Quantity of food f that can be stored at n in season s of year y	Parameter
a_{zfsy}	Intercept of the demand curve for food f for consumers in adaptation zone z during season s of year y	Parameter

b_{zfsy}	Slope of the demand curve for food f for consumers in adaptation zone z during season s of year y	Parameter
Q^{U}_{zfsy}	Quantity of food f consumed in adaptation zone z during season s of year y	Primal variable
$Q^{W\to U}_{znfsy}$	Quantity of food f bought by consumers in adaptation zone z from the market n during season s of year y	Primal variable
$\underline{Q^{U}_{zfsy}}$	Consumption limit for food f in adaptation zone z during season s of year y (to model export)	Parameter

Mathematical Formulation

We describe the mathematical formulation below split up by different activities. Parentheses identify dual variables. For KKT conditions, duals follow the complementary slackness condition.

1. Crop producer

$$max \sum_{c,s,y} df_y \left(\sum_{n} Q^{F\to D}_{zncsy} \pi^{D\to F}_{ncsy} - C^{F}_{zcsy} A_{zcsy} \right) - \sum_{c,s,y,n} \left(\frac{1}{2} C^{chg}_{zsy} \left(A_{zcsy} - A_{zcs(y-1)} \right)^2 \right)$$

subject to

$$Q^{F\to D}_{zncsy}, A_{zcsy}, Q^{F}_{zcsy} \geq 0$$

$$\textstyle\sum_c A_{zcsy} = A^{Total}_{zs} \qquad (\delta^1_{zsy})$$

$$Q^{F}_{zcsy} = Y_{zcsy} A_{zcsy} \qquad (\delta^2_{zcsy})$$

$$Q^{F\to D}_{zncsy} = \Psi_{zn} Q^{F}_{zcsy} \qquad (\delta^{18}_{zncsy})$$

The KKT conditions corresponding to this optimization problem are as follows.

$$\delta^2_{zcsy} - \textstyle\sum_n \Psi_{zn} \delta^{18}_{zncsy} \geq 0 \qquad (Q^{F}_{zcsy})$$

$$\delta^1_{zsy} + df_y \left(C^{F}_{zcsy} + C^{chg}_{zsy} A_{zcsy} + C^{chg}_{zs(y+1)} A_{zcsy} \right) \geq 0 \qquad (A_{zcsy})$$

$$-df_y\left(C_{zsy}^{chg}A_{zcs(y-1)}+C_{zs(y+1)}^{chg}A_{zcs(y+1)}\right)-$$

$$\delta_{zcsy}^{2}Y_{zcsy}$$

$$\delta_{zncsy}^{18}-df_y\pi_{ncsy}^{D\to F} \geq 0 \qquad (\delta_{zcsy}^{2})$$

2. Livestock producer

$$max\sum_{s,y}df_y\left(\sum_{n,\xi}Q_{znsy}^{C\to D,\xi}\,\pi_{nsy}^{D\to C,\xi}-C_{zsy}^{C}Q_{zsy}^{C}\right)$$

subject to

$$Q_{zsy}^{sl},Q_{zsy}^{C},Q_{zsy}^{C,\xi} \geq 0$$

$$Q_{zsy}^{C} = (1+r_{birth}-r_{death})Q_{zs(y-1)}^{C}-Q_{zs(y-1)}^{sl} \qquad (\pi_{zsy}^{C})$$

$$Q_{zsy}^{sl} \geq kQ_{zsy}^{C} \qquad (\delta_{zsy}^{9})$$

$$Q_{zsy}^{C} \geq Q_{zsy}^{C,Herd} \qquad (\delta_{zsy}^{10})$$

$$Q_{zsy}^{C,milk} = Y_{zsy}^{milk}Q_{zsy}^{C} \qquad (\delta_{z(milk)sy}^{2})$$

$$Q_{zsy}^{C,beef} = Y_{zsy}^{beef}Q_{zsy}^{sl} \qquad (\delta_{z(beef)sy}^{2})$$

$$Q_{znsy}^{C\to D,\xi} = \Psi_{zn}Q_{zsy}^{C,\xi} \qquad (\delta_{zn\xi sy}^{18})$$

The KKT conditions corresponding to this optimization problem are as follows.

$$df_yC_{zsy}^{C}-\delta_{z(milk)sy}^{2}Y_{zsy}^{(milk)}-\delta_{zsy}^{4}+kd_{zsy}^{9}-$$
$$d_{zsy}^{10}-(1+r_{birth}-\ r_{death})\pi_{zsy}^{C} \geq 0 \qquad \left(Q_{zsy}^{C}\right)$$

$$\delta_{z\xi sy}^{2}-df_y\Psi_{zn}\delta_{zn\xi sy}^{18} \geq 0 \qquad (Q_{zsy}^{C,\xi})$$

$$\delta_{zsy}^{4}-\delta_{zsy}^{9}-\delta_{z(beef)sy}^{2}Y_{zsy}^{beef}+\pi_{zs(y+1)}^{C} \geq 0 \qquad \left(Q_{zsy}^{sl}\right)$$

$$\delta_{zn\xi sy}^{18}-df_y\pi_{nsy}^{D\to C,\xi} \geq 0 \qquad \left(Q_{znsy}^{C\to D,\xi}\right)$$

3. Distributor

$$max \sum_{f,s,y} df_y \left\{ \sum_n \left(Q_{nfsy}^{D_s} \pi_{nfsy}^{W\to D} - Q_{nfsy}^{D_b} \pi_{nfsy}^{D\to F} \right) - \sum_r C_{rfsy}^{D} Q_{rfsy}^{D} \right\}$$

subject to

$$Q_{nfsy}^{D_b}, Q_{nfsy}^{D_s}, Q_{rfsy}^{D} \geq 0$$

$$\sum_{r\in R_{in}^n} Q_{rfsy}^{D} + Q_{nfsy}^{D_b} = \sum_{r\in R_{out}^n} Q_{rfsy}^{D} + Q_{nfsy}^{D_s} \qquad \left(\delta_{nfsy}^{6}\right)$$

$$\sum_f Q_{rfsy}^{D} \leq \underline{Q_{rsy}^{D}} \qquad \left(\delta_{rsy}^{16}\right)$$

The KKT conditions corresponding to this optimization problem are as follows.

$$df_y \pi_{nfsy}^{D\to F} - \delta_{nfsy}^{6} \geq 0 \qquad \left(Q_{nfsy}^{D_b}\right)$$

$$\delta_{rsy}^{16} + df_y C_{rfsy}^{D} + \delta_{s_r fsy}^{6} - \delta_{d_r fsy}^{6} \geq 0 \qquad \left(Q_{rfsy}^{D}\right)$$

$$\delta_{znfsy}^{6} - df_y \pi_{nfsy}^{W\to D} \geq 0 \qquad \left(Q_{nfsy}^{D_s}\right)$$

4. Storage Operator

$$\sum_{y,h,n,f} df_y \left(\pi_{nfsy}^{U\to W} Q_{nfsy}^{W_s} - \pi_{nfsy}^{W\to D} Q_{nfsy}^{W_b} - C_{nfsy}^{W} Q_{nfsy}^{W} \right)$$

subject to

$$Q_{nfsy}^{W_b}, Q_{nfsy}^{W_s}, Q_{nfsy}^{W} \geq 0$$

$$Q_{nf(sy-1)}^{W} + Q_{nfsy}^{D_b} = Q_{nfsy}^{W} + Q_{nfsy}^{D_s} \qquad \left(\delta_{nfsy}^{11}\right)$$

$$Q_{nfsy}^{W} \leq \underline{Q_{nfsy}^{W}} \qquad \left(\delta_{nfsy}^{8}\right)$$

The KKT conditions corresponding to this optimization problem are as follows.

$$df_y \pi_{nfsy}^{U\to W} - \delta_{nfsy}^{11} \geq 0 \qquad \left(Q_{nfsy}^{W_b}\right)$$

$$C_{nfsy}^{W} + \delta_{nfsy}^{8} + \delta_{nfsy}^{11} - \delta_{nf(sy+1)}^{11} \geq 0 \qquad \left(Q_{rfsy}^{D}\right)$$

$$\delta_{znfsy}^{11} - df_y \pi_{nfsy}^{U\to W} \geq 0 \qquad \left(Q_{nfsy}^{W_s}\right)$$

Here $sy-1$ is the previous season (which could be in the previous year) and $sy+1$ is the next season (which could be in the next year).

5. Consumer

$$\sum_{f,s,y} df_y \left(a_{zfsy} Q^U_{zfsy} - \frac{1}{2} b_{zfsy} {Q^U_{zfsy}}^2 - \sum_n \pi^{U\to W}_{nfsy} Q^{W\to U}_{znfsy} \right)$$

subject to

$$Q^U_{zfsy}, Q^{W\to U}_{znfsy} \geq 0$$

$$\textstyle\sum_n Q^{W\to U}_{znfsy} = Q^U_{zfsy} \qquad (\delta^{19}_{zfsy})$$

$$Q^{W\to U}_{znfsy} \geq \Psi_{zn} Q^U_{zfsy} \qquad (\delta^{17}_{znfsy})$$

$$Q^U_{zfsy} \leq \underline{Q^U_{zfsy}} \qquad (\delta^{20}_{zfsy})$$

The KKT conditions corresponding to this optimization problem are as follows.

$$df_y\left(b_{zfsy} Q^U_{zfsy} - a_{zfsy}\right) + \delta^{18}_{zfsy} + \delta^{20}_{zfsy} + \textstyle\sum_n \Psi_{zn} \delta^{17}_{znfsy} \geq 0 \qquad (Q^{W\to U}_{znfsy})$$

$$df_y \pi^{U\to W}_{nfsy} - \delta^{19}_{zfsy} - \delta^{17}_{znfsy} \geq 0 \qquad (Q^U_{zfsy})$$

6. Market clearing

$$\sum_z Q^{F\to D}_{znfsy} = Q^{D_b}_{nfsy} \qquad (\pi^{D\to F}_{nfsy})$$

$$Q^{D_s}_{nfsy} = Q^{W_b}_{nfsy} \qquad (\pi^{W\to D}_{nfsy})$$

$$Q^{W_s}_{nfsy} = \sum_z Q^{W\to U}_{znfsy} \qquad (\pi^{U\to W}_{nfsy})$$
